# Working from home during COVID-19: boundary management tactics and energy resources management strategies reported by public service employees in a qualitative study

**DOI:** 10.1186/s12889-024-18744-y

**Published:** 2024-05-07

**Authors:** Laura Seinsche, Kristina Schubin, Jana Neumann, Holger Pfaff

**Affiliations:** University of Cologne, Faculty of Human Sciences & Faculty of Medicine and University Hospital Cologne, Institute of Medical Sociology, Health Services Research and Rehabilitation Science, Chair of Quality Development and Evaluation in Rehabilitation, Eupener Str. 129, 50933 Cologne, Germany

**Keywords:** COVID-19, Working from home, Energy management, Boundary management, Work-home conflict, Work delimitation, Exhaustion, Well-being, Public service

## Abstract

**Background:**

Increased working from home has imposed new challenges on public service employees, while also granting opportunities for job crafting. Grounding on the Job Demands-Resources model and Hobfoll’s Conservation of Resources theory this exploratory research aims to investigate the work-nonwork balance of employees one and a half years after the outbreak of the COVID-19 pandemic. Therefore, the research focus lies on employees’ job crafting strategies to optimize their working from home experience concerning boundary management and energy resource management.

**Methods:**

Twelve semi-structured telephone interviews were conducted with public service employees from different sectors in Germany. The experiences were content analyzed using the software MaxQDA and inductive and deductive categories were derived.

**Results:**

Boundary management comprised different strategies such as communicative (e.g., negotiating work time), physical (e.g., going to the garden), temporal (e.g., logging off in between the work day) and behavioral (e.g., prioritizing tasks) strategies. The job crafting strategies regarding energy management included preventing exhaustion (e.g. taking breaks), healthy cooking and energy management in case of sickness (e.g. deciding on sick leave).

**Conclusions:**

This qualitative case study enriches research on job crafting by offering insights on boundary tactics and energy resources management strategies for remote working during the COVID-19 pandemic. The results point out different starting points for employees and decision makers, how a work-nonwork balance, energy management and thus employees’ wellbeing may be increased when working from home in the future.

**Trial registration:**

The study design and methodology were approved by the Ethics Committee of the University of Cologne and the study was prospectively registered (Ref No. 21-1417_1).

**Supplementary Information:**

The online version contains supplementary material available at 10.1186/s12889-024-18744-y.

## Background

The COVID-19 pandemic has expedited the shift of work processes towards a remote working setup in Europe [1]. Consequently, this has resulted in a collision between conventional work-life boundaries and new work demands and resources that employees had to face [2]. Especially for German public service employees working from home (WFH) was new during the pandemic [3, 4]. Moreover, the transition to WFH has, for numerous individuals, particularly during periods of isolation or partial confinement, entailed a complete blending of work and personal life, as well as the necessity to accept the loss of role boundaries [5]. The blurring of work and non-work can have negative impacts on employees’ mental health [6–8]. When WFH during the pandemic, public service employees reported work-related fatigue due to blurred boundaries and work-home conflict [9] as well as increased stress and pressure [10] associated with high work demands. However, at the same time job resources such as work autonomy and increased time flexibility are provided in a flexible WFH environment [11, 12]. This freedom in work design can be used by employees to proactively craft their jobs by adjusting the working conditions to their needs and thus support the handling of high work demands [13, 14]. Hence, this study aims to investigate strategies of public service employees handling WFH. More specifically, we aim to investigate strategies for boundary management and energy resources management. For this purpose, we need to draw on a broad theoretical foundation that is introduced in the following sections.

First, the Job Demands-Resources model (JD-R model) is introduced briefly as a general framework for this study. Since the change to WFH brought about a change in the work conditions of public service employees, the JD-R model, as an established model in the scientific community, provides a helpful perspective for the analyses of job demands, job resources and their impact on employees’ health. Second, we focus on job crafting strategies as an integrative part of the JD-R model and argue for a particular emphasis on strategies targeting the management of boundaries and energy during WFH as a research interest. Third, the Conservation of Resources (COR) theory is introduced as it provides a valuable link between the two different job crafting strategies boundary management and energy resources management.

## Job demands-resources model (JD-R model) and job crafting

The JD-R model divides job characteristics in two factors, namely job demands and job resources [15, 16]. While job demands consume energy and are related to psychological costs, job resources refer to motivational aspects of the job that are functional to achieve goals or reduce psychological costs such as support of colleagues or job autonomy [15]. The model is widely used [17] and can explain the impact of job demands on employees’ health on a long-term and short-term basis through a health impairment process. Furthermore, job resources can buffer the negative health effects of job demands [17, 18]. In relation to the WFH environment described above, higher job demands may have caused work-related fatigue, while blurred boundaries can be seen as job demands and increased job autonomy as a job resource that may be able to buffer job demands.

Later, job crafting has been integrated in the JD-R model. Job crafting is a “proactive behavior through which employees change their work environment and is more specifically conceptualized as strategies that individuals use to shape their job characteristics (i.e., job demands and resources) to regulate their motivation and energy level” [19, p. 457]. There are three categories to classify adaptive strategies to cope with high work demands [19–21]: (a) dealing with depleted resources (e.g., coping strategies, recovery); (b) work and non-work boundary management (e.g., segmentation); and (c) altering job characteristics (e.g., job crafting). All these strategies have been integrated by de Bloom et al. [13] in an integrative needs model of job crafting separating the different goals, motives and dimensions of job crafting. Psychological needs as motives for job crafting can be either approach or avoidance needs [22]. Avoidance needs concentrate on reducing physical or psychological strain and are based on the desire to avoid a negative state. In order to minimize strain and the following exhaustion, employees can therefore seek recovery in form of detachment, stress reduction or relaxation [13, 23, 24]. According to the model of de Bloom et al. [13], there are job crafting strategies that secure optimal functioning for employees at work. Dealing with depleted resources and boundary management are named as such strategies. Boundary management is situated at the interface between the home and work domain, while energy management strategies can also be applied during work time. Since employees work from home, a focus on the interface between both domains and securing optimal functioning during the new situation with excessive WFH seems promising as a research interest.

Thus, in this study we will focus on strategies that target (a) dealing with depleted resources (energy resources management) and (b) support boundary management between work and non-work (boundary management). In the following the two job crafting strategies and their outcomes are presented in detail, before the connection between COR theory and the JD-R model is established. Then the aim and research questions of the study are explained.

### Boundary management, energy resources management and outcomes

In general, job crafting behavior has been found to be a protective factor for employees’ mental health [25] due to the reduction of psychological distress [22, 26], burnout [27, 28], and exhaustion [29, 30]. For an overview of the positive outcomes of job crafting on employees’ health and mental health such as effects on well-being, resilience, vitality, reduced fatigue, reduced distress [13, 14]. According to de Bloom et al. [13] other studies that have investigated job crafting at the work interface level focused on boundary crafting behaviors [31, 32], work–family integration strategies [33, 34] and boundary work tactics [35]. Especially, the later qualitative study by Kreiner et al. developed categories to classify boundary tactics, namely behavioral (e.g. using other people, leveraging technology), temporal (e.g. controlling work time), physical (e.g. manipulating physical space) and communicative tactics (e.g. setting expectations). These categories will be used to classify boundary tactics of the interviewed public service employees during the COVID-19 pandemic. Results of other studies have shown, that involuntarily working more from home can lead to blurred boundaries [34] and a segmentation between work and non-work could be a useful strategy for employees to protect their wellbeing [33, 36, 37].

Several studies have highlighted the benefits of various strategies for relaxation and recovery from work stress (for a review of recovery research, s [38, 39]). Specifically, engaging in physical activity has been shown to promote detachment from work and enhance relaxation levels [38, 40, 41]. Taking rest breaks is also effective in preventing fatigue and maintaining employee performance levels [42, 43]. The quality of these breaks is enhanced when employees have control over their activities and engage in what they prefer [44, 45]. Spending time in nature or outdoors is identified as one of the most effective methods for recuperating from job stress [46]. Furthermore, Bennett et al. [47] discovered that support from supervisors in recovery can help employees mentally distance themselves from work more easily. Moreover, employees have the potential to experience recovery while working through work-related strategies (such as checking emails) or taking micro-breaks (like having a snack) [48, 49]. According to a study of Fritz et al. [48] the five most common micro-breaks that were not work-related were: “(1) drink some water, (2) have a snack, (3) go to the bathroom, (4) drink a caffeinated beverage, and (5) do some form of physical activity including walks or stretching” (p. 33). Research by Op den Kamp et al. [50] indicates that individuals can actively regulate their physical and mental energy levels, and that engaging in such self-management can enhance their work performance.

### Conservation of resources (COR) theory

The COR theory by Hobfoll explains that individuals can only utilize limited resources (e.g. motivation, time, energy), which they have to distribute over their life domains [51]. Thus, job crafting can help to conserve resources by reduction or elimination of job demands that deplete their resources. Additionally, it can expand valuable resources and lead to an optimized resource management. For example, in a WFH environment gained time flexibility enables employees to adjust the work day start and end times to accommodate both work-related and non-work-related demands. In turn, this can support employees to optimize their recovery from work [52]. In the JD-R model the buffer effect of job resources on job demands, that can decrease exhaustion, relates to the COR theory where the (anticipated) loss of resources results in experienced stress. Furthermore, the COR theory concurs with the boundary theory [53–55]. It proposes that employees should separate life domains – especially the border between work and non-work. The underlying idea is similar to the COR theory in the way, that resources are limited. Thus, borders allow humans to strike a balance between the demands of different domains in order to prevent exhaustion and foster wellbeing [31, 54].

### Aim and research questions

Based on these broad theoretical perspectives, we argue that the COVID-19 pandemic has led to an increase of WFH, where boundary management and energy management may have become more important. Since WFH was mandatory during certain phases of the COVID-19 pandemic, this situation is ideal to gain more knowledge about applied boundary management and energy management when WFH. To prevent exhaustion, proactively engaging in job crafting behaviors such as boundary management and energy resources management might be the key to enhance employees’ wellbeing when WFH during the COVID-19 pandemic and in the future. This study stands out because it specifically examines public service employees, a group not extensively covered in existing literature [56, 57]. Its qualitative approach is valuable as it provides in-depth, contextual insights, particularly important in understanding the impacts of the shift to WFH on this workforce segment. To our knowledge, strategies of public service employees to deal with WFH have been researched in a qualitative approach in Australia and the Philippines [58, 59]. However, research investigating the link between recovery experiences and job crafting activities on boundary management is still scarce [60, 61].

Due to the COVID-19 development and increased WFH we sought to generate more knowledge about public service employees’ strategies to improve their work-home balance and optimize energy levels. Therefore, we investigate the tactics employees utilize to manage the interface between work and nonwork. Additionally, we are interested in strategies that support employees to replenish their energy during WFH. The underlying idea is, that employees need functioning boundary management tactics in order to be able to refill their energy levels. The special situation during the pandemic made WFH mandatory for many employees, albeit employees had the choice to WFH during less restricted phases of the pandemic. However, the mostly mandatory character of WFH should be considered in this study. Hence, we derived the following research questions:

1. How do public service employees manage boundaries between work and home life when working from home during the COVID-19 pandemic?
2. How do employees manage their energy levels when working from home during the COVID-19 pandemic?

## Methods

The same sample and procedure were also used in our article analyzing job crafting behaviors of public service employees during COVID-19 [62], in which a different theoretical focus, namely time-spatial job crafting (s [63]). was applied. In the former study, the sample and procedure are described in detail according to the qualitative reporting guidelines (COREQ) by Tong et al. [64]. In the following, the methodological approach is briefly summarized.

### Study design

Our research employed a qualitative method to explore our research questions, aligning with the interpretivist paradigm. This paradigm suggests that reality is a social construct shaped by individuals who assign meanings to their experiences, perceiving the social world through these constructs [65, 66]. Therefore, we used problem-centered interviews to grasp the social reality from the perspectives of individuals, focusing on their perceptions, actions, and thought processes concerning a specific topic, while maintaining an unbiased stance [67]. Given the scarcity of existing research on this subject [68–70], an exploratory approach was considered suitable. This approach is likely to yield rich and detailed insights into the experiences and strategies of public service employees who work from home [71]. The study design and methodology were approved by the Ethics Committee of the University of Cologne and the study was prospectively registered (Ref No. 21-1417_1).

### Participants and procedure

All of the participants provided their email address and consent to participate in further studies during a web-based survey on WFH in Spring 2021. Hence, they were contacted for this interview study in autumn 2021. We applied a purpose sampling strategy aiming at achieving maximal variation in our sample [72]. Sampling criteria were gender, age, leadership position and current job position. Additionally, the duration of WFH in the agency and participants’ perception of how their agency implemented WFH was taken into consideration. One exclusion criterion was applied, sorting out employees who could not or only partially complete their tasks at home. The final sample is depicted in Table 1.

All of the interviewees were invited via email, in which they received information regarding the study. After they provided their informed consent, they were invited to provide dates to schedule the telephone interviews.

**Table 1:** Characteristics of the interviewed public service employees

Inter-view	Age	Gender	Field of agency	Number of employees in agency	Work experience in agency (yrs.)	Leadership position	Start of WFH	WFH amount (days/week)	Fixed work-place (WFH)
1	59	m	building and real estate	ca. 180	31	n	March 2020	5	y
2	61	f	construction industry	4.000	28	y	2016	5	y
3	60	f	district governance	7 (in unit)	14	y	March 2020	0 (before 4–5)	y
4	50	m	data protection	ca. 175	3	y	before COVID-19	3	y
5	62	f	social welfare	1.100	36	n	Spring 2020	ca. 4	y
6	53	m	IT service	300 (in department)	2	n	March 2020	5	y
7	62	m	building and property management	2000	16–17	y	ca. 2009	1–3	y
8	49	m	environmental management	1200	20	n	March 2020	3–4	y
9	29	f	learning and education	1000	1	n	April 2021	4–5	y
10	56	m	information and statistics	300	10	y	March 2020	5	y
11	55	m	customs	10 (in unit)	12	y	2019	4	y
12	55	m	telecommunication	3000	4	y	March 2020	5	y

### Data collection

The semi-structured interview guideline (see Additional File 1) was developed on the basis of a prior quantitative study [73]. Beside a first warm-up question, the interview guideline encompassed four main topics on work organization, leadership and collaboration, scope of action and health. Each topic was opened with a narrative impulse question and invited participants to share their experiences with WFH. The guideline was handled flexibly, giving the interviewees the opportunity to include their own topics and maintaining narrative flow. Nevertheless, the semi-structured guideline ensured a certain comparability between the interviews [67, 72]. The interviews were carried out from December 2021 to February 2022 by three researchers (L.S., K.S., J.N.) via telephone due to the COVID-19 related social restrictions. During the data collection only the research team was present and field notes were taken. On average the interviews lasted between 26 and 60 min. For the analysis the audio-taped interviews were transcribed verbatim by a professional transcription service.

### Data analysis

Data was analyzed using qualitative content analysis by Kuckartz [74] and the software MAXQDA 2022 (VERBI GmbH, Berlin, Germany) [75]. Several coding rounds were applied, where initially the first author developed a preliminary category system. Then the second author (K.S.) commented and revised the categories. In a second coding round all of the interviews were coded by the first author and then discussed until the coding scheme was finalized. Deductive categories were derived from literature (e.g. “communicative” or “temporal boundary tactics” by Kreiner et al. [35]), while new inductive categories were formed from the data material.

The interview quotes were translated from German into English by the first author. All categories and example quotes can be found in Additional file 2.

## Results

**Fig. 1:**
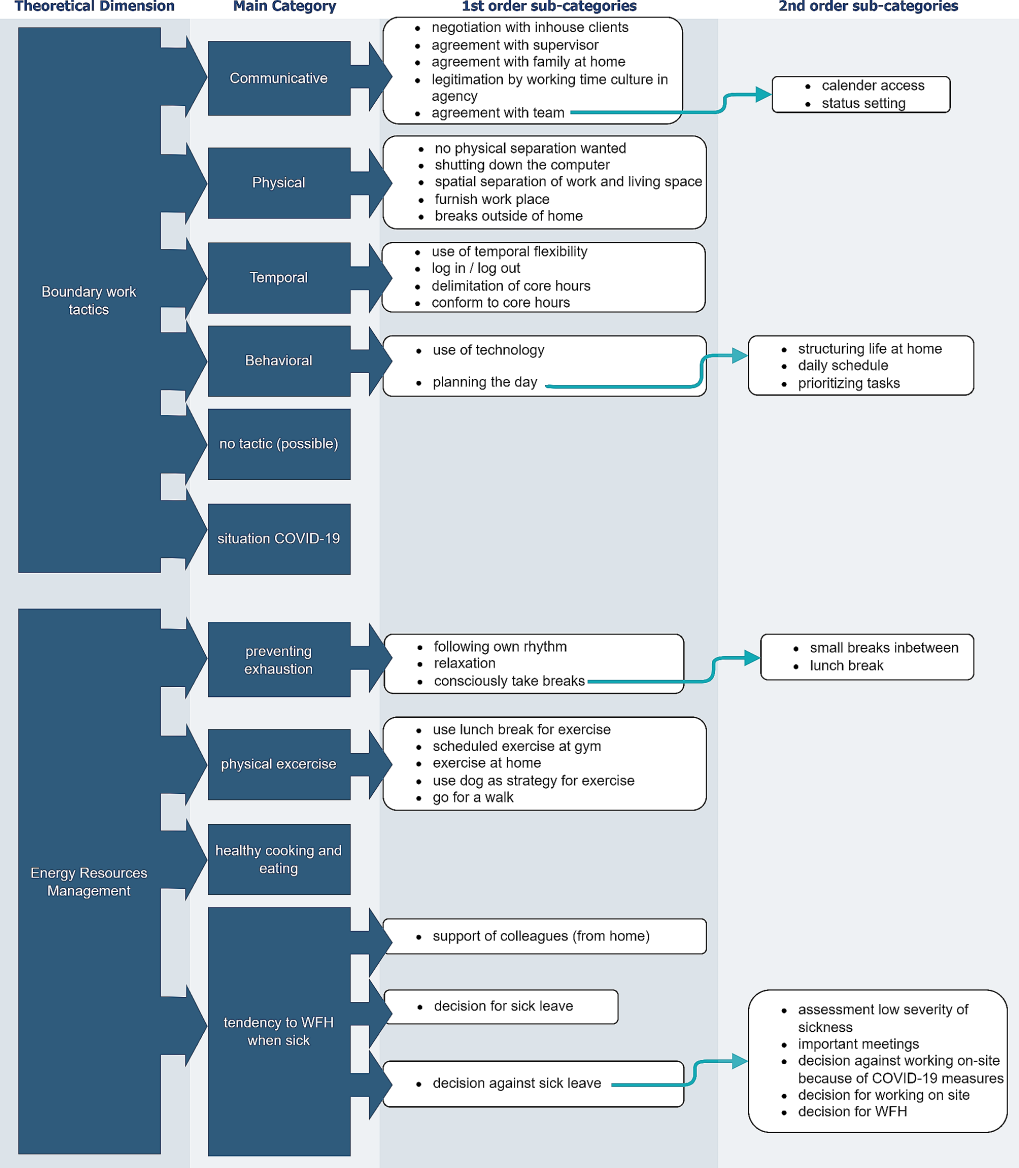
Overview of the main and sub-categories

In the following the results are reported in the order of the deductive categories on boundary work tactics and energy management. To provide a clear overview, we created a diagram with the main categories and sub-categories (Fig. 1).

### Boundary work tactics

Public service employees reported to utilize different strategies to craft their work-nonwork balance when WFH. In the following, the strategies of the sub-categories “communicative”, “physical”, “temporal”, “behavioral”, “no tactic” and “situation COVID-19” are being reported.

### Communicative

The communicative crafting strategies relate to the management of peoples’ expectations in regard to possible boundary violations [35]. The public service employees had different communication partners, with whom expectations had to be managed. First, there were work relations with other colleagues or inhouse clients. One interviewee stated that inhouse colleagues “*place an order so late […], you have no choice but to call them the next morning*” (interview 5). Therefore, the negotiation starts after placing the order to manage the required working time and set the work boundaries.

Besides the communication with other employees, the legitimation by culture played a role. Thus, the working time culture in a certain agency can shape the work boundaries:

*“…the usual agreements - no e-mails after 8 p.m. and none before 6 a.m. - that is the agreement with us*.” (interview 12).

A work culture with fixed rules regarding work-related availability may be able to prevent pressure and the delimitation of core hours. Especially, employees that seek a clear boundary management style may be attracted by work cultures offering guidelines. Additionally, one employee explained that the transition to a home-based work environment during lockdown stages of the COVID-19 pandemic had no impact on work culture:

“*It’s a business, you know when you’re working and most of the customers or press people or people involved know exactly when you’re where. It’s not something that changes overnight.”* (interview 7).

In this case, the transition to working from home did not change the work culture and remained the same. Moreover, the interviewee mentions that the habits of an agency do not change overnight and it is known by other colleagues, when a person can be contacted at best. In interview 1 the role of the supervisor is brought up, as a means to find an agreement regarding working time:

*“…because I have (.) personally enforced for myself, I have decided, enforced and agreed with my employer, (.) how my working time is structured.”* (interview 1).

Within teams, arrangements took place such as team times that had been agreed upon (s. interview 12). Another way was to openly communicate break hours and linked availability to the team:

*“So I can actually regulate it quite well when I work from home and I have also briefed my colleagues so far, that they know I always take a long lunch break and I have already found some imitators, exactly.”* (interview 9).

This brief insight indicates that new work habits limiting work boundaries were formed also for the entire team, when WFH. Technology supported the communicative boundary tactics as calendar access could help team members to see, on which days someone was present at the office (s. interview 4). Furthermore, the status in the collaborative software Microsoft teams or Skype was used to show availabilities (s. interview 5). One interviewee stated:

*“It’s actually the case that everyone goes online in the morning and then you can indicate on Skype that you’re now available in green or that you’re now at work, that you’re in red or that you’ll be right back in yellow or something. But I have to say that most people forget that.”* (interview 11).

Even when setting the status was simple, it was still not flawless as the employee mentioned that “*most people forget that*”.

Another boundary tactic concerned the communication with family. One interviewee mentioned that his spouse worked in the same agency and thus shared the same flexible working conditions. Therefore, it was easy for them to divide chores and alternate who “*walks the dog at lunchtime*” (interview 6).

### Physical

According to Kreiner et al. (2009) physical boundary tactics can be used to manipulate physical space in form of creating or reducing physical distance to the work place or using items (e.g. calenders, photos) to integrate or separate the domains. The public service employees reported boundary tactics concerning the physical aspects of the work environment when WFH. For some it was the action to “*close the computer and call it a day*” (interview 9), while others needed a strict separation of work and living space:

*“And that’s different than when you’re in the office. You leave the house in the morning, then you’re at work, then you can completely block out home and when you finish work and come home again, you’re back in your private life.”* (interview 1).

When WFH, interviewee 9 found an adapted physical strategy for WFH days:

*“I make myself a cup of coffee, have breakfast, and now I try to separate things strictly. So that I really do have breakfast first and then sit down in my study to start things off separately, so to speak. So that I don’t start reading the first emails during breakfast, exactly.”* (interview 9).

On the contrary, for some employees the physical separation does not seem to be necessary at home:

*“I like to work at the dining table, but only when it’s clear that I’ll be alone, I am alone all the time. I also don’t like to spread everything out and then I have to somehow put it away again so that I can continue working upstairs.”* (interview 3).

Similarly, interviewee 6 explained that he has no physical separation of working and private life, but rather *“set [his office on site] up very privately”.*

A physical strategy also encompassed to get away from the work place in order to spend breaks outside of home. For example, interviewee 8 commented:

*“If I have my lunch break at home, I can go shopping in the meantime, I can go for a walk anywhere, but just also take a walk to another agency or go to the doctor or things like that.”* (interview 8).

Sometimes employees made use of a physical strategy in order to have a clear transition phase. Thus, one employee explained how she went to the bakery instead of commuting (s. interview 9).

### Temporal

Temporal boundary tactics encompass the controlling of work time by manipulating schedules (e.g. banking time from one domain to be used later) or removing oneself from work/home for a specific amount of time [35]. Regarding temporal boundary management tactics there were different characteristics. Thus, employees made use of temporal flexibility that was given within usual working hours:

*“And that might be easier to plan, if I work from home and I could simply offer support in a more self-determined way that you can have a sick child brought over to you or something and then somehow postpone the work a bit.”* (interview 3).

As interviewee 3 suggested a postponement of work, the use of time flexibility was closely linked to taking small breaks or blocking off time from work. In this regard one employee stated:

*“You’ve logged out, right? That is not working time. And it is permitted to work between 6:30 a.m. and 8:00 p.m. and the number of interruptions and the duration of the interruptions do not matter.”* (interview 6).

For some employees temporal flexibility advanced to a total delimitation of core hours:

*“That means that under certain circumstances I still check e-mails or something at 10 p.m. and possibly also still answer*.” (interview 3)”.

One employee in a leadership position reported that she “*would be available on weekends as well”* (interview 2).

For a higher control of work-life boundaries and working time, interviewee 8 made a decision:

*“And now there quite consciously to say, I for myself, go away from these times and try to use again the classic work time, between 9 and 18 o’clock, as I did it earlier in the office also, hands tied.”* (interview 8).

### Behavioral

Behavioral tactics describe the use of technology or other people’s skills to facilitate boundary work and prioritizing the demands of either work or home domain [35]. Employees utilized different behaviors to ensure their work-life boundary tactics that were either based on technological equipment or planning their day by structuring and prioritizing tasks. When using technology, one employee reported to set the phone to flight mode to be not available (s. interview 1). Interviewee 6 explained that logging off the system is not the same as being unavailable for calls:

*“Whereas the logging in and out, that’s purely a time recording thing. I can log out and theoretically still be available. If I don’t want to be available, then I have to set our communication tool accordingly. I can then set an “absent mode” where no calls reach me, right?”* (interview 6).

Besides using communication technology, employees planned their work days to have a structure:

*“That it is clear what I have to do today. I think it is important for working from home to plan the day: That it’s also clear, when I’m going to stop working. So that the danger does not exist, that one/ It is important that one, I find, that one sets a beginning and an end point for work and then also takes a break and plans the day accordingly.”* (interview 3).

The setting of priorities was also mentioned when WFH:

*“One must also organize and structure oneself at one’s workplace. Perhaps also to set priorities.”* (interview 1).

As for the private tasks, there may be less coordination needed, as one was at home, if *“the parcel delivery guy comes or when the chimney sweep makes an appointment”* (interview 6).

### No tactic and Situation COVID-19

Even though employees have developed their own strategies to handle WFH and blurred work-life boundaries, there were situations, where strategies could simply not be applied:

*“But as I said, you’re just at home and the doorbell rings once in a while. Then someone comes who wants something from you, who brings you something or delivers something, or, or, or. So you have to mentally switch back and forth a bit.”* (interview 1).

The blur of boundaries could not always be prevented as it occurred unforeseen. One of the interviewees also reported lacking a strategy:

*“And then it was often the case that this saved working time [due to omission of commuting] was instead converted into office work, i.e. real work in front of the computer. In other words, I worked more.*” (interview 8).

For him the saved working time resulted in more work hours. In other cases, the COVID-19 pandemic has led to the delimitation of work hours:

*“It already starts with all these extensions of the work time, that I write emails on Saturdays or at ten in the evening, [that] was due to this special situation [COVID-19] and I think we all agree that we don’t want that.”* (interview 8).

Besides the above reported results one employee mentioned an employer-initiated boundary tactic. In this case the agency shut off the mail server between 8 pm and 6 am to prevent employees sending emails late at night (interview 7). For the sake of completeness, we included this circumstance in this paper, but the focus will remain on employee-initiated boundary strategies.

### Energy resources management

The results indicate, that public service employees used different strategies to manage their energy levels when WFH. The four main sub-categories were “preventing exhaustion”, “physical exercise”, “healthy cooking and eating” and “tendency to WFH when sick”.

### Preventing exhaustion

One factor that helped employees to prevent exhaustion when WFH was the ability to follow their own rhythm. This included to start the work day according to their own needs:

*“Yes, so early bird, that’s not my thing at all, right? So, and now I can also reconcile that better with work than when I am at the office.”* (interview 12).

Another strategy to prevent exhaustion was to integrate time for relaxation. One of the employees mentioned that she took a power nap at home, if she felt exhausted:

*“So, now I’m going to do a half hour power nap, lay down on my bed and really get away from it all, and then I’m also fitter.”* (interview 3).

Besides sensing the body’s need for rest, taking a conscious break could refill depleted energy levels. In this manner interviewee 1 stated regarding lunch break:

*“I consciously take a lunch break at noon.”* (interview 1).

The lunch break may have been also used for other activities to leave thoughts of work behind, such as:

*“I kind of go out in the garden and take my break there, raking leaves or something depending on the season or I sit in the sun for half an hour or I go to the mailbox.”* (interview 5).

Additionally, the public service employees took time for small breaks during their work day. These breaks could be handled more flexibly (s. interview 10). Interviewee 9 explained her working strategy of dividing tasks and work time by inserting small breaks:

*“So it’s just simple/ Well, I personally have the feeling that I can simply divide my time more freely. I can say in a much more relaxed way, I’ll do this task now, then I’ll do that task, then I’ll do the next task, and if I need another ten-minute break, I’ll go out on the balcony and get some fresh air.”* (interview 9).

### Physical Exercise

Some public service employees used their lunch break to do physical exercise. The physical exercise helped them to replenish their energy for the rest of the work day. For example, interviewee 11 reported:

*“And because I can now work from home, I use this break for my exercise, which I used to only be able to do in the evening when I was at home. And now I do it at lunchtime and almost every lunchtime. And then I’m logged off for an hour and a half or two hours. And then I continue to work afterwards. And that’s actually a good thing, because then you’re fit again, at least that’s how it is for me.”* (interview 11).

Other employees integrated an exercise at the gym in their weekly work plan (s. interview 1), whereas working at home also offers an opportunity for exercise. Therefore, online meetings can be attended while standing up or even moving around the house:

*“And apart from that, I find the fact that I can move around when I want to move around, not sitting in a WebEX session - if I do it standing up, I’m much more mobile, more agile and that’s pleasant.”* (interview 12).

Similarly, one employee reported that she used phone calls to integrate physical exercise in her work day instead of sitting at her desk:

*“… someone calls and you talk on the phone and then of course you walk a bit. You walk around the house and look out of the window or get yourself a glass of water or something.”* (interview 5).

For other employees having a dog worked as a strategy for exercise, since the dog needed to be taken outside regularly (s. interview 6, interview 2) and others just went for a work during lunch break (s. interview 8).

### Healthy cooking and eating

When WFH the lunch break can be used to prepare fresh food. Thus, one interviewee reported that she experienced a healthier life style when working at home than being on business trips:

*“But now when you’re at home like that, you can make yourself a cauliflower soup and make yourself a salad or something like that and eat, I think, healthier.”* (interview 2).

### Tendency to WFH when sick

When employees felt sick, their energy levels may have been low and they adapted their working habits or strategies accordingly. By presenting a case and asking the following question (“You notice cold symptoms in yourself. Would you go to work?”), we prompted employees to find out, how and why their decisions varied about calling in sick, when they worked from home. We separated the answers in the following main categories: “support of colleagues”, “decision for sick leave” and “decision against sick leave”. The support of colleagues was treated as a separate aspect, while the initial decision for employees seemed to be, if they should take a sick leave or not. When they decided against sick leave, there were different reasons and motivations to work - whether working on site or WFH.

First, the aspect to be available for the support of colleagues was a consideration, whether one stayed at home with or without sick leave. For one employee that was simply a matter of collegiality:

*“And of course, everyone has a telephone with them, even if they are ill, and can answer a call if someone wants to know something or a colleague wants to know where to find something. But that has nothing to do with duty, it has to do with collegiality.”* (interview 7).

Second, the decision for sick leave seemed to depend on the individual feeling and assessment of the illness. Therefore, interviewee 6 explained, where he would draw a line. If he felt too weak to sit down, even at the desk at home, he would call in sick:

*“I felt like I was coming down with the flu, right? I was at least a little weak and dull and noticed that when you lie down, you feel better than when you sit or stand. So then I would not have sat down at the desk.”* (interview 6).

Another strategy was to continue WFH and assess the sickness over the course of days. In this manner one employee decided to withdraw himself from work, *“if it doesn’t get better after two, three days […] then cure it by calling in sick*” (interview 1).

Third, the decision against sick leave encompassed a variety of reasons. Some provided reasons were special to the situation when working at home or on site. Initially, the severity of sickness also seemed to be an indicator for the decision to work:

*“So now if I have a little bit of a cold and a little bit of a cough and maybe a sore throat, but no headache or aching limbs, I would work.”* (interview 9).

As for working on site one interviewee stated, that he also went to work because of waiting tasks:

*“But with a slight cold I went to work, yes because I wanted to get my work done.”* (interview 11).

Therefore, the measures to prevent the spread of COVID-19 acted as a barrier for employees to show up to work with cold symptoms:

“*Because I would probably go to work with it, but there is the clear announcement in the current time that even with slight cold symptoms we must not come to the office.”* (interview 4).

Before the pandemic, employees may have gone to work with a cold. One of the employees stated that he had his own office in the company and thus *“would also have gone to work [before the pandemic]”* (interview 6).

Except for the COVID-19 prevention measures, there were other reasons that public service employees referred to, when explaining WFH while being sick. WFH seemed to function as an alternative for a sick leave:

*“In the past, you could have alternatively just taken a sick leave. You wouldn’t have been able to work from home. And now, I think, if you’re in such a floating state, okay, you have the feeling that you’re not actually sick, but you also don’t want to be suspected of infecting others, then you just work from home at that moment.”* (interview 5).

Especially, the means to take care of oneself were different when employees worked from home. Interviewee 8 gave an insight on his strategy:

*“That means this “I’m just going to check something” and I can decide for myself whether I’m going to sit there for half an hour and just briefly check emails or whether I’m actually going to sit down at the computer for four, five, six hours.”* (interview 8).

The adjustment of the work day according to the own feeling of the health state was possible when WFH. Similarly, interviewee 11 told that he took a nap in between, which was also only possible at home, while one employee reported, that WFH offered more opportunities to treat oneself:

*“Working from home gives you much better opportunities to treat certain types of colds, for example. For example, I could inhale much more easily here or things like that, you know? I can actually do that while working from home and still work. And you can’t usually do all these things as well or at all in the office. And that’s a difference, yes.”* (interview 1).

## Discussion

The aim of this study was to generate more knowledge about public service employees’ strategies to improve their work-home balance and optimize energy levels while WFH. This qualitative study provides several key learnings: (1) The study contributes to the growing body of literature of real WFH experiences during COVID-19 pandemic. (2) It provides valuable insights to boundary management tactics and energy management of employees. (3) The results offer practical guidance for employers and employees, how to optimize WFH conditions in the future. (4) The study provides implications for further research in determining effective WFH strategies. In the following, the research questions are answered.

### How do public service employees manage boundaries between work and home life when working from home?

Similarly to the results of Kreiner et al. [35] the results indicate that public service employees utilized behavioral, temporal, communicative and physical boundary work tactics when WFH. Regarding behavioral strategies no signs were found for the category “using other people” [35], whereas “leveraging technology” was an implemented strategy when WFH. Additionally, “planning the day” was - referring to the WFH setting - rather implemented by creating a daily schedule or prioritizing tasks. Thus, more sub-categories were formed for public service employees when WFH.

Temporal boundary strategies (“controlling work time”, “finding respite”, [35]) were present strategies in the sample. In the study’s sample, employees can utilize little breaks, including those as brief as a lunch break, on a daily basis. Concurring with the findings of Kreiner et al. [35], the temporal removal from work could be used with a physical tactic such as getting a physical distance (e.g. using lunch break for a walk).

Within the communicative tactics, different people (e.g. supervisor, team, family) played a role. The management of expectations and finding agreements with the team was essential in this category. During WFH there is an interesting interplay between the categories “communicative”, “temporal” and “behavioral”. Public service employees utilize technology to regulate their work-related availability, which is a behavioral strategy. Simultaneously, the use of collaborating software such as Microsoft teams serves as a means to communicate their availability to team members and co-workers. Thus, setting a status implies a communicative function; it signals *“that I’m available: Aha, I’m already there now”* (interview 5) such as the turning on/ turning off the office light on site. Simultaneously, it imposes new challenges, since the participants stated, that co-workers often forget to change their status. Forgetting to indicate the status can either result in not being seen as available or being reached during leisure time. The last may result in working overtime or work life delimitation, if borders are not protected. “Protecting private time” is a strategy that has been found to significantly influence employees’ subjective wellbeing [31]. Furthermore, in the WFH setting “log in/ log out” can be part of a temporal boundary strategy. If the non-availability is communicated, it allows employees to withdraw from work for a certain amount of time such as a lunch break used for exercise.

Physical tactics that are used, depend on the want of employees to integrate or separate life domains [35]. The former authors state that individuals create their own ideal level of work-home integration or segmentation. In the same regard, the experiences of the interviewees show different styles of wanted integration or segmentation of the work and home domain. For some it is important to have a physical border between these two life domains, while others enjoy temporal flexibility and even voluntarily tend to work life delimitation in their physical space. The two categories “no tactic” and “situation COVID-19”, which showed that boundary work tactics could not be applied, add to the existing main categories under the circumstances of mandatory WFH during this period. When WFH, the boundaries between work and private life are blurred to a massive extent, since the physical separation is lacking. Therefore, strategies may exist to handle boundaries, but in unpredicted situations such as the ring of the doorbell, the plans of employees to separate domains are disrupted. These situations are challenging for employees who prefer a separation of life domains.

Working conditions that may support successful boundary management are team culture and work hours culture, if there is a well-established limit of work time. These results are consistent with other studies that suggest the framing of boundaries can be socially shared and norms can be established [76].

It is proposed, that a reciprocal relationship exists between the boundary tactics [35]. This kind of relationship can be found in the communicative and behavioral tactics as the communication of boundaries or setting the phone to flight mode automatically reduces the challenge of boundary management. Likewise, the temporal removal from the work space for breaks serves as one of the energy management strategies that are presented below. In the following the link between boundary tactics and energy resources management will be discussed drawing on the theoretical background.

### How do employees manage their energy levels when working from home?

Public service employees reported various strategies to either minimize exhaustion, integrate health behaviors during their work day or how they dealt with their energy level during sickness. Other studies found microbreaks to be effective in replenishing energy levels [49]. Similarly, public service employees found ways to withdraw from work by inserting small breaks, mainly in form of physical activity such as going to the mailbox or on the balcony. Nevertheless, physical exercise was reported in a separate category, since employees had many ways to replenish their energy through exercise. Physical activity in particular leads to detachment from work and high levels of relaxation [38, 40, 41]. Rest breaks can prevent fatigue and help to sustain the performance level of employees [42, 43]. In the same manner employees report that they start fresh into the second half of the work day after their lunch break or exercise (s. interview 11, interview 5). The use of the temporal flexibility and short periods of withdrawing from work serves not only as a temporal boundary style. For example, a small break from work might work as a combined strategy as it (1) refills the energy level and (2) serves as a moment of leaving thoughts of work behind, if the break is taken outside on the balcony and (3) simultaneously is a physical boundary tactic as one physically steps out of the working area.

This example concurs with COR theory [51] and boundary theory [53–55] as employees can conserve their resources by taking a break and withdrawing temporarily from work and striking a balance between work and non-work domain. If both theories are taken into account, boundary management needs to be applied at the right time in order to keep individuals from suffering under exhaustion. Boundary management tactics show, how the employees manage the borderline between WFH and living at home, whereas the energy management strategies go beyond the mere handling of borders, but can explain in which regard employees apply strategies to keep their energy reserves and stay healthy during WFH. Additionally, other studies refer to the protective function of job crafting for employees’ mental health, e.g. by reducing exhaustion [25, 29, 30]. If the categories are integrated in the JDR model, the blurred work and life domains impact employees as a job demand when WFH, while boundary management and energy resources management can function as strategies to reduce exhaustion. Therefore, job resources such as autonomy or support from colleagues (e.g. if private time is agreed on and protected), can buffer the negative effect on employees’ health. Consequently, they can minimize the anticipated loss of resources, that results in stress according to COR theory.

WFH is able to provide more autonomy as a job resource and control for employees to spend their breaks according to their preferences. For experiencing a deep relaxation and recovery, it is crucial that employees experience control during their break and engage in a preferred activity [44, 45]. One of the most effective ways to recover from job stress seems to be spending time in nature or outdoors [46], which may also be easier to achieve when WFH such as walking the dog or just sitting in the garden (s. interview 2, interview 6).

The tendency to work from home while sick was bound to employees’ own assessment of severity of the illness. From employees’ perspectives, WFH has been an alternative to being on sick leave, because it offers the opportunity to take care of the body’s need for rest. For example, alternatives to medication or a nap could be taken in between the work day. In this case, boundary management tactics that allow a temporal removal from work are simultaneously used with strategies to replenish employees’ energy levels. Another link is proposed through the work-related availability, even when employees are sick. Here the boundary is permeable to support colleagues. However, these practices suggest the emergence of a culture of presenteeism in remote work settings, where employees continue working even when ill [77]. Working on-site may act as a safeguard, not only by allowing clearer boundaries and reducing the need for extended availability, but also by protecting employees from the risks of presenteeism that come with WFH [78]. In a traditional workplace, managers can step in and send unwell employees home, fulfilling their duty of care. In contrast, when working remotely, monitoring of employees is more challenging, placing greater reliance on individual responsibility. Employees who work from home while sick prioritize their work over their health, a phenomenon known as interested self-endangerment [79]. The blurring of work-life boundaries in a home office setting increases health risks, including self-endangerment and mental strain [80], while also enabling employees to make use of different resources to care for oneself.

### Recommendations for future research

In this paper we identified boundary work tactics that employees use when WFH and therefore expanded the understanding of boundary management during the COVID-19 pandemic. We found specific tactics that may be useful to optimize WFH arrangements. Additionally, the findings provide insights into the possible connections between the categories and combined strategies (e.g., behavioral and temporal boundary tactics or temporal boundary tactics and energy management) when WFH. Further research could explore how boundary strategies interact with each other and potentially reinforce one another, leading to the development of a model to better understand these dynamics. Further research could also investigate problematic areas, where boundary tactics fail or focus on specific preferences such as work-home integration or segregation [35].

Linking our findings to the JD-R model and Hobfoll’s COR theory, a strong boundary management may support conserving resources and using them for leisure. Thus, employees may experience a better recovery experience, if they have the energy to exercise even after a full work day and stay healthy in the long term. To our knowledge, research investigating the link between recovery experiences and job crafting activities is still scarce [60].

More studies investigating the relationships between leisure, health and successful boundary tactics and energy management are necessary to determine, when the line between work and non-work should be drawn to maximize positive effects for employees and employers. Therefore, an additional testing of the relationships, the theoretical framework and the transferability of results to other groups of employees or sectors would be appropriate by using quantitative research methods. Moreover, boundary tactics and energy management should be investigated more thoroughly in hybrid work settings to identify the most effective working conditions in terms of available job resources, job demands, utilized job crafting strategies and an optimized recovery experience. Since WFH had a mandatory character in our study, research on hybrid work settings can provide a more differentiated picture of possible job crafting strategies in different professions. Diary studies could be used to measure stress indicators and exhaustion as well as daily job crafting strategies to define individual types and an effective handling of WFH.

In their qualitative study on archetypes of sickness attendance, Ruhle and Süß [81] discovered that employees have become more sensitive regarding working on site because of the COVID-19 measures, but still they are not inclined to call in sick when they are at home. More research in form of larger quantitative samples or longitudinal designs will be necessary to fully understand these habits and underlying culture. Therefore, a closer look at interested self-endangerment when WFH will be helpful as well. In addition, larger case studies could explore different types of organizational culture (supporting/hindering boundary management or interested self-endangerment behaviors).

### Recommendations for practice

The study provides insights into employees’ individual boundary and energy management styles. These experiences may support other employees in finding their own valuable strategy to manage the blurring of work-life boundaries or staying vital and active when WFH. Furthermore, employers should offer information to raise employees’ awareness regarding these possible strategies and health benefits that derive from the application of boundary work tactics and energy management strategies. Bennett et al. [47] found out, that employees can easily mentally distance from work, when supervisors support their recovery. If supervisors take care of themselves, this self-care behavior also may be adapted by employees, since supervisors act as role models for health promoting behavior [82]. Similarly, supervisors should provide job resources and encourage employees to take microbreaks [49] as this strengthens the rest break intention as well [42].

Another approach is to train employees in applying these work-related strategies [49], strengthen their skills such as time management or self-regulation or to enable employees to negotiate their work time or work-family arrangements more freely [33]. The reported employer measure to shut off the e-mail server appears to be a last resort of protection of employees’ recovery time and health. Instead, a corporate culture, where the communication and management of boundaries is possible and encouraged in the team and organization should prevent those drastic measures.

The tendency to work from home when sick, shows that employees were much more sensitized to not go to work because of the COVID-19 prevention measures. At the same time, they are inclined to not always call in sick, but work from home even if they are ill. Here it is important that employees are trained in health literacy, which can be seen as prerequisite for self-care behavior [83]. They need to be able to manage their energy resources and apply strategies, because only if they know the bottom of their resources and know their own body well, this strategy of self-assessment can work out when employees become sick. Additionally, supervisors need to be sensitive in order to look out for employees so employees do not wear themselves out, when they are WFH while sick. In this regard, it is important for employers to see employees face-to-face from time to time and thus be able to assess their state of health.

### Strengths and limitations

Strengths of the study are the close orientation to literature findings and theoretical grounding of the empirical findings. However, concerning the qualitative findings a number of limitations must be considered. These findings are not universally applicable to other groups or settings (outside of public service employees in certain agencies in Germany), indicating the need for more quantitative studies or additional qualitative case studies in diverse business environments. The sample size and composition also present constraints, as many participants were over 50 years old and without children at home. Comparing the sample with the demographic features of public service employees in 2021, 42.3% of public service employees in Germany were between 45 and 59 years old and represent a major part of the professional group [84]. Hence, the age group represents a large portion of the public service employees. Interestingly, around 57% of the public service employees are women, but they are underrepresented in our study. One possible explanation is that only 46% of women serve in the higher service and 36% have a leadership position [84]. This suggests more investigation is needed since boundary management tactics can differ, examining various age groups is essential, particularly considering potential work-family conflicts and the home environment. There is also a possibility of selection bias in choosing interview participants, as those who volunteered may have preferred WFH and may have been more interested in the subject. Furthermore, the interviews were conducted at a specific time, capturing only the perspectives of public service employees WFH one year after the COVID-19 pandemic began. A notable strength of the study is the involvement of three researchers in conducting and coding the interviews, which helps mitigate subjective bias. The use of qualitative reporting criteria and a semi-structured interview guide ensured a standardized process. Moreover, the research is based on the JD-R model and Hobfoll’s widely recognized COR theory, adding value to the existing body of knowledge. The findings offer insights into boundary management tactics and energy resource management strategies for remote working during the COVID-19 pandemic. This qualitative study identifies patterns and provides directions for future research aimed at improving the work conditions and health of public employees WFH.

## Conclusions

This qualitative case study enriches research on job crafting by offering insights on boundary tactics and energy resources management strategies for remote working during the COVID-19 pandemic. Findings reveal that public service employees developed personal crafting strategies to cope with boundary management and energy resources management when WFH, including physical, behavioral, communicative or temporal strategies. Strategies aiming at energy resources management included preventing exhaustion, physical exercise and also managing WFH when employees were sick. Drawing on the JDR model and Hobfoll’s COR theory, this qualitative study identifies patterns and various opportunities and risks for health when WFH - aspects that are particularly relevant as remote work is likely to remain in the future. Furthermore, it provides directions for future research and practice aimed at enhancing the work conditions and wellbeing of public employees WFH.

### Electronic supplementary material

Below is the link to the electronic supplementary material.

Supplementary Material 1

Supplementary Material 2

## Data Availability

The datasets generated and/or analyzed during the current study are not publicly available due to the personal information and sensitive information from employers and employees, comprehended in the interviews and which could in theory be might be traced back to individual respondents. The original data is only available on site after contact with the corresponding author on reasonable request, to ensure data access complies with the procedures of the General Data Protection Regulation (GDPR). All information analyzed during this study is included in this published article and its supplementary information files.

## Abbreviations

COR: Conservation of resources theory
JD-R: Job Demands-Resources model
WFH: Working from home
