# Supplementary material for: Working from home during COVID-19: boundary management tactics and energy resources management strategies reported by public service employees in a qualitative study
Source: BMC Public Health. 2024 May 7;24:1249. doi: 10.1186/s12889-024-18744-y (PMC11075362; doi:10.1186/s12889-024-18744-y)
Supplement: Supplementary file 1 — Supplementary Material 1 [file 12889_2024_18744_MOESM1_ESM.pdf]

Additional File 1 | Interview guideline questions.

Guiding Questions	Sub-Questions
**Warm-Up Question**	
Please tell me briefly how the introduction of WFH went in your agency.	- Certain regulations on the part of the agency?<br>- How were these coordinated?<br>- Will WFH remain in the future?
**Organization of Work**	
Please tell me what a typical day looks like, when you work from home.	- Do you stick to a certain daily routine?<br>- Have you found your own personal approach to the daily work routine when WFH?<br>- When and how are you available for colleagues?<br>- What has helped you to organize your work when WFH?
In what way can you see differences to the way you work when you were present at the office?	- Have you found that you work different hours than when you were present at the office?<br>- Has your workload changed?<br>• Unchanged/increased/decreased?
Please tell me about the difficulties you experienced at the beginning when WFH.	-What has improved this now?<br>- What has helped you?
**Scope of Action**	
Please tell me about the freedom you have in completing your work tasks when WFH.	- What do you like about your work? (What motivates you?)<br>- To what extent do you have time or performance constraints?<br>- To what extent are mistakes tolerated? (Control)<br>- How much responsibility are you given?
To what extent do you notice differences compared to your freedom/ scope for action in presence?	- To what extent do you feel pressure to perform? (more/less)<br>- Is the work more/less controlled?<br>- ((work intensification?))
**Leadership and Collaboration**	
Tell me how you perceive the interaction between managers and employees in your agency.	- How often are you in contact/communication with your manager?<br>- How is the communication with your manager in the home office?<br>• Does the need for discussion come from you or does the manager approach you?<br>- How do you experience recognition and appreciation at your workplace? (on the part of colleagues, on the part of the manager)<br>- In what form does your manager express criticism? How do you deal with it? (work, person)
To what extent can you identify differences in the way managers and employees deal with each other when they are present?	- How does behavior transfer to the WFH situation?
Example: Do you have the feeling that there are colleagues in your organization who intentionally send emails very early or very late, which was not common before WFH?	- Did you notice something in this way?<br>- What do you think is the reason for this? (e.g. recognition performance, commitment)
**Health**	
Please tell me how working from home affects your health.	- Positive/ negative?<br>- Why is it so?<br>- Physical problems (e.g. tension, fatigue, headaches)<br>- Mental problems (balanced, full of energy vs. feeling anxious, distracted)<br>- Life satisfaction?
How was it for you in presence?<br>(Can you notice any differences in your physical or mental health?)	- Positive/ negative?<br>- Why is it so?<br>- Physical problems (e.g. tension, fatigue, headaches)<br>- Mental problems (balanced, full of energy vs. feeling anxious, distracted)

	- Life satisfaction?<br><br>- If applicable, what is the biggest advantage of the home office for you?
<u>Case</u>: You notice cold symptoms in yourself. Would you go to work?	- How would you act when WFH?<br>- How would you act when working in presence?<br><br>- Why yes / Why not?

Finally, is there anything you would like to add that you have not yet mentioned?
