# Supplementary material for: Working from home during COVID-19: boundary management tactics and energy resources management strategies reported by public service employees in a qualitative study
Source: BMC Public Health. 2024 May 7;24:1249. doi: 10.1186/s12889-024-18744-y (PMC11075362; doi:10.1186/s12889-024-18744-y)
Supplement: Supplementary file 2 — Supplementary Material 2 [file 12889_2024_18744_MOESM2_ESM.pdf]

Theoretical Dimension	Main category	Sub-category	Sub-category	Definition	Quotes	Coding rules
**Boundary work tactics**				Kreiner, Hollensbe and Sheep (2009) classified four types of boundary work tactics (behavioral, temporal, physical, and communicative) that individuals utilized to help create their ideal level and style of work-home segmentation or integration.		
	**Communicative**			Managing expectations in advance of a work-home boundary violation (e.g., stating preferences to parish or family ahead of time) (Kreiner, Hollensbe, Sheep, 2009)		
		negotiation with inhouse clients			Well, this happens to us from time to time, in relation to the entire department, that we find it impertinent that people still place an order so late when there is no one left who can accept it at all or say anything about it, and then you have no choice but to call them the next morning. (interview 5)	
		legitimation by working time culture in agency			It's a business, you know when you're working and most of the customers or press people or people involved know exactly when you're where. It's not something that changes overnight. (interview 7)<br><br>...the usual agreements - no e-mails after 8 p.m. and none before 6 a.m. - that is the agreement with us. (interview 12)	
		agreement with supervisor			...because I have (.) personally enforced for myself, I have decided, enforced and agreed with my employer, (..) how my working time is structured. (interview 1)	
		agreement with team			So I can actually regulate it quite well when I work from home and I have also briefed my colleagues so far, that they know I always take a long lunch break and I have already found some imitators, exactly. (interview 9)<br><br>But otherwise we have also arranged team times, right? So in the morning before eight actually not at all... (interview 12)	
			calendar access		that we maintain our calendars in Outlook in such a way that it says on which days I am present at the office and on which days I am working from home. (interview 4)	
			status setting		It's like that, you don't have to set it, but you can and I always do that, that I set myself to green when I'm available, so to speak. That usually means that when I log in in the morning, they see, automatically, that I'm available: Aha, I'm already there now. (interview 5)<br><br>It's actually the case that everyone goes online in the morning and then you can indicate on Skype that you're now available in green or that you're now at work, that you're in red or that you'll be right back in yellow or something. But I have to say that most people forget that. (interview 11)	Coded, if the log in or log out process has a communicative function to colleagues (status is shown)
		agreement with family at home			My wife sits across from me when we work from home. She has the same employer and the same working conditions, thus we can alternate quite well. So alternately everyone walks the dog at lunchtime. (interview 6)	

Theoretical Dimension	Main category	Sub-category	Sub-category	Definition	Quotes	Coding rules
	**Physical**			Managing physical artifacts, manipulating physical space (Kreiner, Hollensbe, Sheep, 2009)		
		no physical separation wanted			I like to work at the dining table, but only when it's clear that I'll be alone I am alone all the time. I also don't like to spread everything out and then I have to somehow put it away again so that I can continue working upstairs. (interview 3)	
		shutting down the computer			And then the workday continues, depending on when I start working again in the afternoon, until around, well, half past five, six, and then I close my computer and call it a day. (interview 9)	
		spatial separation of work and living space			And that's different than when you're in the office. You leave the house in the morning, then you're at work, then you can completely block out home and when you finish work and come home again, you're back in your private life. (interview 1)<br><br>I make myself a cup of coffee, have breakfast, and now I try to separate things strictly. So that I really do have breakfast first and then sit down in my study to start things off separately, so to speak. So that I don't start reading the first emails during breakfast, exactly. (interview 9)	
		furnish work place			Yes, because I also had an office for myself on site, except for the external consultant who was there from time to time, but otherwise I didn't have to ensure that I cleaned my desk in the evenings or anything like that, right? At that time, before the pandemic, that was also basically my office and I also set myself up very privately there. (interview 6)	
		breaks outside of home			If I have my lunch break at home, I can go shopping in the meantime, I can go for a walk anywhere, but just also take a walk to another agency or go to the doctor or things like that (interview 8)<br><br>That's why I sometimes do it in such a way that in the morning I somehow, I don't know, go to the bakery to pick up rolls or something. Then you already feel like you're on your way to work, which does you a world of good. (interview 9)	
	**Temporal**			Controlling Work Time: Manipulations of one's regular or sporadic plans (e.g., banking time from home or work domain to be used later, blocking off segments of time, deciding when to do various aspects of work)<br><br>Finding Respite: Removing oneself from work- home demands for a significant amount of time (e.g., vacations, getaways, retreats) (Kreiner, Hollensbe, Sheep, 2009)		
		use of temporal flexibility			And that might be easier to plan, if I work from home and I could simply offer support in a more self-determined way that you can have a sick child brought over to you or something and then somehow postpone the work a bit. (interview 3)	Coded, if employees make use of temporal flexibility within limits of usual working hours
		log in / log out			You've logged out, right? That is not working time. And it is permitted to work between 6:30 a.m. and 8:00 p.m. and the number of interruptions and the duration of the interruptions do not matter. (interview 6)	Coded, if log in our log out is used to remove oneself from work and making use of temporal flexibility
		delimitation of core hours			But, yes, so I would be available on weekends as well. (interview 2)<br><br>That means that under certain circumstances I still check e-mails or something at 10 p.m. and possibly also still answer. (interview 3)	Coded, if employees extend usual work hours, e. g. choose to work in evening hours or at the weekend
		conform to core hours			And now there quite consciously to say, I for myself, go away from these times and try to use again the classic work time, between 9 and 18 o'clock, as I did it earlier in the office also, hands tied. (interview 8)<br><br>That's why I've largely kept to the times I usually work in the office, yes. So that's actually the case, there's not that much difference. (interview 10)	Coded, if employees reported that they did not extend their work hours to unusual times

Theoretical Dimension	Main category	Sub-category	Sub-category	Definition	Quotes	Coding rules
	**Behavioral**			Using other people (skills), leveraging technology (voicemail), prioritizing tasks (Kreiner, Hollensbe, Sheep, 2009)		
		use of technology			Whereas the logging in and out, that's purely a time recording thing. I can log out and theoretically still be available. If I don't want to be available, then I have to set our communication tool accordingly. I can then set an "absent mode" where no calls reach me, right? (interview 6)<br><br>The phone is then also set to flight mode. (interview 1)	
		planning the day	structuring life at home		There's a difference between having to do everything in the evening when you get home, whether it's hanging up the laundry and cleaning out the dishwasher. Or whether you can always do it during the day so that it runs in the background and you just save time, or that you're just at home when the parcel delivery guy comes or when the chimney sweep makes an appointment, so you don't have to coordinate it, you're just there, right? (interview 6)	
			daily schedule		That it is clear what I have to do today. I think it is important for working from home to plan the day: That it's also clear, when I'm going to stop working. So that the danger does not exist, that one/ It is important that one, I find, that one sets a beginning and an end point for work and then also takes a break and plans the day accordingly. (interview 3)	
			prioritizing tasks		One must also organize and structure oneself at one's workplace. Perhaps also to set priorities. (interview 1)	
	**no tactic (possible)**			There were no employee-initiated strategies reported or situations, where tactics could not be applied.	But as I said, you're just at home and the doorbell rings once in a while. Then someone comes who wants something from you, who brings you something or delivers something, or, or, or. So you have to mentally switch back and forth a bit. (interview 1)<br><br>And then it was often the case that this saved working time (due to ommission of commuting) was instead converted into office work, i.e. real work in front of the computer. In other words, I worked more. (interview 8)<br><br>We also have a company agreement that protects colleagues who are not in management positions from too much work by turning off the servers from 8 p.m. to 6 a.m., which means that emails sent or received are recorded on the server but not forwarded to the mailboxes. This is an agreement with the staff council, so that colleagues are not still working on emails until 10 or 11 pm. (interview 7)	
	**situation COVID-19**				It already starts with all these extensions of the work time, that I write emails on Saturdays or at ten in the evening, [that] was due to this special situation [COVID-19] and I think we all agree that we don't want that. (interview 8)	

Theoretical Dimension	Main category	Sub-category	Sub-category	Definition	Quotes	Coding rules
**Energy Resources Management**				Employees proactively manage their vitality (i.e., physical and mental energy) to promote optimal functioning at work. The findings suggest that peoplewho engage in Proactive Vitality Management may sometimes use work-related strategies and micro-breaks at work.<br>(Op den Kamp et al., 2018)		
	**preventing exhaustion**					
		following own rhythm			Yes, so early bird, that's not my thing at all, right? So, and now I can also reconcile that better with work than when I am at the office. (interview 12)	
		relaxation			So, now I'm going to do a half hour power nap, lay down on my bed and really get away from it all, and then I'm also fitter. (interview 3)	
		consciously take breaks	lunch break		I consciously take a lunch break at noon (interview 1)<br><br>I kind of go out in the garden and take my break there, raking leaves or something depending on the season or I sit in the sun for half an hour or I go to the mailbox (interview 5)	
			small breaks inbetween		So it's just simple/ Well, I personally have the feeling that I can simply divide my time more freely. I can say in a much more relaxed way, I'll do this task now, then I'll do that task, then I'll do the next task, and if I need another ten-minute break, I'll go out on the balcony and get some fresh air. (interview 9)<br><br>On the other hand, it is easy to spontaneously insert small breaks in between when working from home. (interview 10)	
	**physical exercise**					
		use lunch break for exercise			And because I can now work from home, I use this break for my exercise, which I used to only be able to do in the evening when I was at home. And now I do it at lunchtime and almost every lunchtime. And then I'm logged off for an hour and a half or two hours. And then I continue to work afterwards. And that's actually a good thing, because then you're fit again, at least that's how it is for me. (interview 11)	
		scheduled exercise at gym			So for example I have a rule that I start my work day later than usual on three days a week because I can easily get to a gym from home in ten minutes (interview 1).	
		exercise at home			Then I start and in between I go downstairs and make myself a coffee or someone calls and you talk on the phone and then of course you walk a bit. You walk around the house and look out of the window or get yourself a glass of water or something. (interview 5)<br><br>And apart from that, I find the fact that I can move around when I want to move around, not sitting in a WebEX session -if I do it standing up, I'm much more mobile, more agile and that's pleasant. (interview 12)	
		use dog as strategy for exercise			As I said, we move around quite a bit at home, even when working from home, because we also check on the dog from time to time and take the dog downstairs or out and let it out into the garden. (interview 6)<br><br>Yes, if I didn't have the dog, I would probably hardly get any exercise - I'll put it that way - because the whole thing that you set out to do and you go jogging or something like that, I can't do it without a dog anyway. Because I have a dog, I definitely walk an average of four kilometers a day, no matter how bad the weather is, how cold or wet (Interview 2)	
		go for a walk			“If I have my lunch break at home, I can go shopping in the meantime, I can go for a walk anywhere, but just also take a walk to another agency or go to the doctor or things like that.” (interview 8)	
	**healthy cooking and eating**				But now when you're at home like that, you can make yourself a cauliflower soup and make yourself a salad or something like that and eat, I think, healthier. (interview 2)	

Theoretical Dimension	Main category	Sub-category	Sub-category	Definition	Quotes	Coding rules
	**tendency to WFH when sick**					
		support of colleagues (from home)			And of course everyone has a telephone with them, even if they are ill, and can answer a call if someone wants to know something or a colleague wants to know where to find something. But that has nothing to do with duty, it has to do with collegiality. (interview 7)	
		decision for sick leave			I felt like I was coming down with the flu, right? I was at least a little weak and dull and noticed that when you lie down, you feel better than when you sit or stand. So then I would not have sat down at the desk. (interview 6)<br><br>So if it doesn't get better after two, three days, a cold like that, I say, then I also go to withraw myself from circulation, so to speak, and then cure it by calling in sick. (interview 1)	
		decision against sick leave	important meetings/tasks		But with a slight cold I went to work, yes because I wanted to get my work done. (interview 11)	
			decision against working on-site because of COVID-19 measures		Because I would probably go to work with it, but there is the clear announcement in the current time that even with slight cold symptoms we must not come to the office. (interview 4)	
			assessment low severity of sickness		So now if I have a little bit of a cold and a little bit of a cough and maybe a sore throat, but no headache or aching limbs, I would work. (interview 9)	
			decision for working on site		I used to go to work with a slight cold or when I had a bit of a cold, right? That's just the limit, at what point you are contagious or are you such a virus slinger that you would actually endanger others and since I also had my own office in the old company and very rarely had customer contact, I would also have gone to work there. (interview 6)	
			decision for WFH		In the past, you could have alternatively just taken a sick leave. You wouldn't have been able to work from home. And now, I think, if you're in such a floating state, okay, you have the feeling that you're not actually sick, but you also don't want to be suspected of infecting others, then you just work from home at that moment. (interview 5)<br><br>That means this "I'm just going to check something" and I can decide for myself whether I'm going to sit there for half an hour and just briefly check emails or whether I'm actually going to sit down at the computer for four, five, six hours.(interview 8)<br><br>But in the year and a half, or two years, I've had that maybe once or twice, that I've said, "Okay, I don't feel well now, but I can work. Then I'll just take a nap, I can do that at home." I can't do that in the office. So that's what I did. (interview 11)<br><br>Working from home gives you much better opportunities to treat certain types of colds, for example. For example, I could inhale much more easily here or things like that, you know? I can actually do that while working from home and still work. And you can't usually do all these things as well or at all in the office. And that's a difference, yes. (interview 1)	
